# Comparison of anticarcinogenic properties of Viburnum opulus and its active compound p-coumaric acid on human colorectal carcinoma

**DOI:** 10.3906/biy-2002-30

**Published:** 2020-10-13

**Authors:** Serdar KARAKURT, Gülsüm ABUŞOĞLU, Zekiye Ceren ARITULUK

**Affiliations:** 1 Department of Biochemistry, Faculty of Science, Selçuk University, Konya Turkey; 2 Department of Medical Laboratory Techniques, Vocational School of Health, Selçuk University, Konya Turkey; 3 Department of Pharmaceutical Botany, Faculty of Pharmacy, Hacettepe University, Ankara Turkey

**Keywords:** Colorectal carcinoma, Viburnum opulus, p-coumaric acid, TP53, BRAF, apoptosis, cell cycle

## Abstract

Resistance to therapeutic agents and the highly toxic side effects of synthetic drugs has spurred new research in the treatment of colon cancer, which has high morbidity and mortality ratios. This study aims to clarify the molecular mechanisms of the anticarcinogenic properties of methanol extract of *Viburnum opulus* L. (EVO)and its main active compound, *trans-p* -coumaric acid ( *p* -CA), on human colon cancer cells (DLD-1, HT-29, SW-620, Caco-2) and healthy colon epithelial cells (CCD-18Co). The effects of EVO on controlled cell death (apoptosis) and the cell division cycle were determined by flow cytometry. Alteration in mRNA and protein expressions of switch genes in colorectal carcinoma (APC, MLH1, TP53, SMAD4, KRAS, and BRAF) were determined by qRT-PCR and Western blot, respectively. Our results show that EVO possesses a strong reducing capacity and free-radical scavenging activity. HPLC analyses prove that *p* -CAis the main compound of EVO. EVO and *p* -CA inhibit the proliferation of human colon cancer cells DLD-1 and HT-29 in a dose-dependent manner. EVO increases apoptosis of DLD-1 cells and halts the cell cycle in the G2 stage in HT-29 cells. mRNA and protein expressions of p53 and SMAD-4 are upregulated, while BRAFs are downregulated. The results were directly proportional to *p* -CA. EVO and *p* -CA up- and downregulate switch genes and protein expressions of DLD-1 cells, which alter the expression of 186 other genes. This is the first study of pharmacological exploration of *V.opulus* in human colon cancer. Its antiproliferative effects may be due to the presence of *p* -CA.

## 1. Introduction

Cancer is one of the leading causes of morbidity and mortalityaround the world. According to the World Health Organization’s 2015 data, the death rate from cancer has reached 8.8 million, which is expected to increase by 70% over the next 20 yearsWHO (2015). Cancer [online]. Website: u1d7f [accessed 30 July 2015].. Colorectal cancer (CRC), seen both in men and women, is the third most common cause of death worldwide (Globocan, 2014). Many factors, including inflammatory bowel disease, colon involvement, epigenetic changes, and family history, are closely related to the progression of CRC (Johnson et al., 2013). Increased age and a lifestyle including high meat consumption, low vegetable consumption, low physical activity, and high alcohol and food consumption are major factors that influence CRC development (Cunningham et al., 2010). Molecular studies showed that CRC is under the control of signaling pathways including Wnt/β-catenin, NF-κB, Nrf2,and PI3K/Akt/mTOR, in which KRAS, BRAF, APC, p53, and SMAD-4 possess crucial importance (Zhang et al., 2015; Dawood et al., 2019; Sheng et al., 2019; Wang et al., 2019). Mutations inAPC, MLH1, TP53, SMAD4, KRAS, and BRAF genes play a crucial role in CRC progression and are a reason for the increased incidence of CRC (Namani et al., 2017). These genes interact with a total of 184 genes; alteration of these genes affects the progression of CRC. 

Several chemotherapeutic drugs including cisplatin have been implemented to treat CRC. Nevertheless, the adverse effects of chemotherapy are connected to high mortality and reactive oxygen species production (Wu et al., 2019).Although surgery is the most effective treatment method, postsurgery chemotherapy or radiotherapy is required because of the high probability of recurrence after surgery. It is necessary to seek new and alternative treatment options to increase the survival rate in CRC. Therefore, identifying a new therapy to contend against CRC is critically important. Plants synthesize hundreds of secondary metabolites as a defense mechanism against insects, fungi, various mammals, and many diseases. However, the molecular mechanisms of those metabolites require more investigation. There is a significant argument for promoting the consumption of vegetables and fruits, which contain plenty of antioxidants that defend against illness and oxidative devastation (Celik et al., 2016; Karakurt et al., 2016). The genus *Viburnum* L., a member of the Adoxaceae family, consists of more than 230 species distributed in the temperate and subtropical regions from South America to Southeast Asia. In Turkey, the *Viburnum* genus is represented by 4 species: *V. lantana* L., *V. opulus* L., *V. orientale* Pall., and *V. tinus* L. *V.opulus* possesses darkred edible fruits containing high amounts of polyphenols such as chlorogenic acid, catechin, epicatechin, and anthocyanins, and organic acids such as ascorbic and oxalic acids (Cam and Hisil, 2007). Thepolyphenols in *V. opulus* have also been used for the synthesis of silver nanoparticles that have been used as an antiinflammatory compound both in vitro and in vivo (Moldovan et al., 2017). The fruits of *V. opulus* have been traditionally used to treat some health problems such as tension, bladder disturbances, duodenal ulcers, cough, cold, kidney, and menstrual and stomach cramps in some countries (Ezov and Koncejev, 2000; Moldovan et al., 2012). It has been demonstrated that *V. opulus* , commonly grownin central Anatolia, has protective functions against urolithiasis and inflammation (Kizilay et al., 2019). *V. opulus* extract presented anticancer properties in Ehrlich ascites tumor-bearing mice (Ceylan et al., 2018). Colonic lesions induced by 1,2 dimethylhydrazine were found to beinhibited by *V. opulus* in the mouse colon (Ulger et al., 2013). However, very few studies have examined the effects of *V. opulus* on human colon cancer. Furthermore, the molecular mechanism of action and the mechanism of cell death have not been elucidated either in vivo or in vitro. Following the o-oxygenation and methylation reactions in plants, hydroxycinnamic acid derivatives including *p* -coumaric and caffeic acid are produced (Russell and Duthie, 2011). These phenolic compounds have a crucial role in the scavenging of free radicals, the regulation of gene and protein expression, xenobiotics metabolism and cellular signaling related to apoptosis, cell proliferation, and the DNA repair system. Anticarcinogenic properties of *p* -coumaric against several human CRC cells such as Caco-2, HT-29, and SW-480 have been investigated in many studies (Janicke et al., 2011). Here, the in vitro anticarcinogenic effects of *V. opulus* and its active compound *p-* CA are compared. 

In this study, we aim to investigate the cytotoxic effect of EVO and *p* -CA on human colorectal cell lines including DLD-1, HT-29, SW-620, and Caco-2 and to clarify the molecular mechanisms.

## 2.Materials and methods

### 2.1. Chemicals

*Trans* - *p* -coumaric acid (55823) was purchased from Sigma (Sigma-Aldrich, St. Louis, MO, USA), and 100 pmol of primers were synthesized by BM Laboratory systems (Ankara, Turkey). CCD-18Co cells (ATCC-CRL-145), modified McCoy’s 5a medium(ATCC-30-2007), EMEM (ATCC-30-2003),RPMI 1640 medium (ATCC-30-2001), and fetal bovine serum (FBS; ATCC-30-2020) were obtained from ATCC (American Tissue Culture Company, Manassas, VA, USA). The SMAD4 (10231-1-AP), KRAS (12063-1-AP), BRAF (20899-1-AP),and TP53 (10442-1-AP) primary antibodies (1/1000 diluted) were purchased from Proteintech (Proteintech Laboratories, Manchester, UK). An apoptosis kit was obtained from BD Biosciences (Woburn, MA, USA). Solvents were HPLC grade; all other chemicals were of analytical grade.

### 2.2. Plant materials

The fruits of *V. opulus* were harvested from a cultivated area in Kayseri, Turkey in September 2017. The plant was identified by the authors, and voucher specimens were placed in the Herbarium of Hacettepe University, Faculty of Pharmacy, Ankara, Turkey under the number HUEF17039. 

### 2.3. Extraction

Fresh fruits (each 50 g) were extracted with methanol 3 times for 3 days and filtered. The concentrated extracts were dried under reduced pressure at 40 °C and lyophilized.

### 2.4. High-performance liquid chromatography (HPLC) analysis

#### 2.4.1. Determination of individual phenolic compounds by HPLC

The phenolic compounds of EVO were analyzed with a Shimadzu LC-20AD HPLC system (Kyoto, Japan). Seventy mg of methanol extract was diluted 40-fold with methanol and filtered through a filter disk for HPLC analysis. Separation of phenolic compounds was accomplished with an Inertsil ODS-3V C18 column (GL Sciences Inc., Tokyo, Japan) (5µm; 4.6 × 250 mm i.d.). The mobile phase flow rate was kept at 1.0 mL/min. Mobile phase A was water containing 0.05% gAc, and mobile phase B was acetonitrile. The gradient conditions were as follows: 0–0.10 min, 8% B; 0.10–2 min, 8–10% B; 2–27 min, 10%–30% B; 27–37 min, 30%–56% B; 37–45 min, 56%–80% B. The temperature of the column was controlled at 30 °C. The injection volume was 20 μL. The phenolic compounds were monitored at 280 nm with an SPD-20A UV-Vis detector. The phenolic compounds in EVO were identified by using authentic standards and comparing with the standard compounds in terms of retention times and UV data. The quantitative analysis of phenolic compounds was performed by use of a calibration curve constructed for each commercial standard, i.e. gallic acid, protocatechuic acid, catechin hydrate, 4-hydroxybenzoic acid, caffeic acid, syringic acid, rutin trihydrate, *trans-p* -coumaric acid, *trans* -ferulic acid, *trans* -resveratrol, *trans* -cinnamic acid, and naringenin. The standard phenolic compounds were dissolved in methanol; 8 different concentrations of each compound ranging from 1 to 65 ppm were prepared by diluting the stock solution. Three injections were performed for each concentration level, and the calibration curves were constructed by plotting the peak area against concentration. The results are expressed as ppm (parts per million).

#### 2.4.2. Determination of organic acids by HPLC

The quantification of organic acids in EVO was carried out with a Shimadzu LC-20AD HPLC system (Kyoto, Japan). Sixty mg of methanol extract was diluted 20-fold with water and filtered through a filter disk for HPLC analysis. A MetaCarb 87H Column (300 × 7.8 mm; Agilent Technologies, Santa Clara, CA, USA) coupled with a MetaCarb 87H Guard Column (50 × 4.6 mm) was used for separation at a flow rate of 0.6 mL/min.The mobile phase was 0.1 N H_2_SO_4_. The organic acids were monitored at 210 nm with the SPD-20A UV-Vis. The organic acids in EVO were identified using the authentic standards and comparing retention times and UV data with the standard compounds. The quantitative analysis of organic acids was achieved by use of a calibration curve constructed for each commercial standard—i.e. citric acid, tartaric acid, maleic acid, succinic acid, formic acid, acetic acid, and fumaric acid. The standard compounds were dissolved in deionized water; 6 different concentrations of each compound ranging from 1 to 100 ppm were prepared by diluting the stock solution. Three injections were performed for each concentration level, and the calibration curves were constructed by plotting the peak area versus concentration. The results were expressed as ppm (parts per million).

### 2.5. Antioxidant activity

#### 2.5.1. DPPH radical scavenging capacity assay

DPPH radical scavenging capacity assay was performed according to a slightly modified version of the method described by Brand-Williams et al. (1995). The extract and the reference, dissolved in methanol at various concentrations (150 µL), were mixed with 1 mM DPPH solution in methanol (50 µL) and incubated for 30 min. The change in absorbance was measured at 517 nm. The DPPH radical scavenging capacity was calculated as given below: 

Inhibition % = [(Ablank−Asample)/Ablank]×100.

Quercetin was used as a reference. Triplicate reactions were performed. The scavenging capacity of the extract was expressed as a half-maximal inhibitory concentration (IC_50_).

#### 2.5.2. Cupric ion reducing antioxidant capacity (CUPRAC) assay

The cupric ion reducing antioxidant capacity of EVO was determined using the method of Apak et al. (2004) with slight modifications. The reaction mixture contained equal amounts of 10 mM of CuCl_2_ solution, 7.3 µM of neocuproine solution, and 1 M of NH_4_Ac buffer solution at pH 7.0. To make the final volume of 200 µL, 25 µL of sample and 25 µL of distilled water were added to the mixture. The final solution was incubated for 30 min at RT (room temperature) and the samples were measured at 450 nm. The result was presented as gallic acid equivalent.

#### 2.5.3. Determination of total phenolic contents

The total phenolic content of EVO was estimated according to the method described by Slinkard and Singleton (1977) using Folin-Ciocalteau’s reagent; 20 µL of extract dissolved in ethanol and 7.5% (w/v) Na_2_CO_3_ solution were mixed with Folin-Ciocalteau’s reagent(1:10). The reaction mixture was incubated at RT for 2 h and measured at 765 nm. The result was presented as gallic acid equivalent.

#### 2.5.4. Determination of total flavonoid contents

The total flavonoid content of EVO was determined by using the aluminum chloride colorimetric method (Chang et al., 2002). Twenty-five µL of thesamplewas mixed with 95% ethanol, 10% AlCl_3_, and 1 M KCH_3_COO. Following 30 min of incubation at RT, the absorbance was measured at 415 nm. The result was presented as quercetin equivalent.

### 2.6. Cell culture

Human CRC cell lines(DLD-1, HT-29, SW-620,Caco-2) and human colon fibroblast cell line CCD-18Co were cultivated in RPMI-1640, McCoy’s 5a, Leibovitz’s L-15, and EMEM growth mediums (ATCC), respectively and supplemented with 2 mM L-glutamine (Sigma-Aldrich), 10% fetal bovine serum (FBS), and 1% penicillin/streptomycin at 37°C in 5% CO_2_ at 90%–95% humidity.

#### 2.6.1. Cell viability assay

1 ×10^4^ cells were seeded into a 96-well plate with growth medium and incubated at 37 °C in 5% CO_2_ for 24 h until the cells attached to the bottom. The cells were then treated with various concentrations of EVO ranging from 1 µg/mL to 1000 µg/mL and *p-* CA ranging from 0 to 1000 µM. The cell viability and proliferation were measured through the Alamar Blue assay (Karakurt and Adali, 2016). The absorbance was measured at 570 nm and 610 nm using a multiwell scanning spectrophotometer (Multiskan Go; Thermo Scientific Co., Waltham, MA, USA). IC_50_ values of EVO were calculated from the sigmoidal plot of inhibition ratio (%) vs. log EVO concentration.

### 2.7. Apoptosis assay

30 × 10^4^of DLD-1 cells were seeded into a 6-well plate and treated with an equivalent IC_50_ concentration of EVO and *p* -CA and incubated for 48 h at 37 °C in 5% CO_2_. Trypsinized and washed (with 10 mM PBS) cells were double-stained with FITC Annexin V and 7-Aminoactinomycin D (7-AAD). The effects of EVO on the percentage of DLD-1 and HT-29 cells which had undergone apoptosis and necrosis were determined according to the manufacturer’s instructions (BD Bioscience, San Jose, CA, USA). Early apoptotic, late-stage apoptotic, or necrotic cells were examined using a NovoCyte flow cytometer (Acea Biosciences Inc, San Diego, CA, USA). 

### 2.8. Cell cycle analysis

15 × 10^4^ of DLD-1 cells were seeded into a 6-well plate and treated with the equivalent IC_50_ concentration of EVO and *p* -coumaric acid, and then harvested by trypsinization. After fixation in 70% ice-cold ethanol for 2 h, the cell pellets were incubated with 100 μg/mL of RNase A and 50 μg/mL of PI for 30 min. Using the NovoCyte flow cytometer, 50,000 cells from each group were subjected to cell cycle analysis.

### 2.9. Preparation of cell lysates and Western blot analysis

30 × 10^4^of DLD-1 cells were seeded into 100 cm2 Petri dishes and treated with the equivalent IC_50_ concentration of EVO and *p* -coumaric acid. Following the incubation, the cells were washed with pre-cold PBS and lysed in RIPA buffer (Cell Signaling Technology, Beverly, MA, USA) with 1 mM of PMSF. The lysates were centrifuged at 14,000 g at 4°C for 15 min. The supernatant was used to determine the protein concentration, which was determined by the BCA method. Fifteen µg of the protein samples were separated by SDS-PAGE (4% stacking gel and 7.5% separating gel), blotted onto PVDF membrane, and incubated with polyclonal (rabbit) anti-APC, anti-TP53, anti-BRAF, anti-SMAD4, anti-KRAS antibodies (Bio-Rad Laboratories, Hercules, CA, USA) overnight at 4°C, and with goat anti-rabbit IgG secondary antibody for 1 h at RT. ECL detection systems (Thermo Scientific Co.) were used for detection. Gels were photographed using the Syngene GBOX Chemi XRQ imaging system (Frederick, MD, USA) and analyzed using the GeneSys software.

### 2.10. Total RNA isolation, cDNA synthesis, and qRT-PCR analysis

The total RNA of DLD-1 cells was extracted using QIAzol Lysis Reagent (Qiagen, Hilden, Germany) according to the manufacturer’s instructions. Quantity and quality of total RNA were measured using an Agilent 2100 Bioanalyzer, and samples with higher RIN (>7) were used for the synthesis of cDNA. cDNA was synthesized from 1 ng of total RNA via reverse transcription using an iScript Reverse Transcription cDNA synthesis kit according to the manufacturer’s instructions (Bio-Rad Laboratories, Hercules, CA, USA). The synthesized cDNAs were subjected to amplification using specific primers that were designed as shown in Table 1. The expression of GAPDH was used as a reference gene, which was used as an internal control for normalization. qRT-PCR was accomplished with a CFX Connect Real-Time PCR Detection System with the optical 96-well plate (Bio-Rad Laboratories). Twenty μL of the reaction mix containing 10 μL SYBR GREEN PCR Master Mix, 2 μL diluted cDNA template, and 0.4 μL of each primer was added into each well. The thermal cycling profile was recommended by the manufacturer: 98 °C for 30 s, 40 cycles at 98 °C for 15 s, 60 °C for 30 s. To confirm the specificity of the primers, melting curves were included after amplification. All reactions were run in triplicate, and the fold-change of mRNA expression was calculated according to the delta delta (2^[−ΔΔCt]^) method.

**Table 1:** Primer sequence of colorectal cancer progression key genes.

Gene	NCBI referencenumber	Sequences (5’à3’)	Annealingtemp. (°C)	PCR product size (base pair)
APC	NM_001127510.3	F- TGAGGCACTGAAGATGGAGA R- TCTGTCCAGAAGAAGCCATAG	60	158
MLH-1	NM_000249.4	F- TGTTAAAGAGGGAGGCCTGA R- CTCACCTCGAAAGCCATAGG	60	157
TP53	NM_001276696.2	F- CACATGACGGAGGTTGTGAG R- TAGGGCACCACCACACTATG	60	158
SMAD4	NM_005359.6	F- CACAGGACAGAAGCCATTGA R- ACGCCCAGCTTCTCTGTCTA	60	150
KRAS	NM_001369786.1	F- TGCAATGAGGGACCAGTACA R- TCCTGAGCCTGTTTTGTGTCT	60	201
BRAF	NM_001374258.1	F- TCAACCACAGGTTTGTCTGC R- TCACTCGAGTCCCGTCTACC	60	164
GAPDH	NM_001357943.2	F-AATCCCATCACCATCTTCCA R-TGGACTCCACGACGTACTCA	60	82

### 2.11. Statistics

Each experiment was repeated at least 3 times. Quantitative data were processed with GraphPad Prism (GraphPad Software v5.0, San Diego, CA, USA). Results are represented as mean ± SD. Statistical comparisons between groups were made using two-way repeated measure analysis of variance (ANOVA) and two-tailed Student’s t-test, where P < 0.05 was considered to be statistically significant.

## 3. Results and discussion

**Figure 1:**
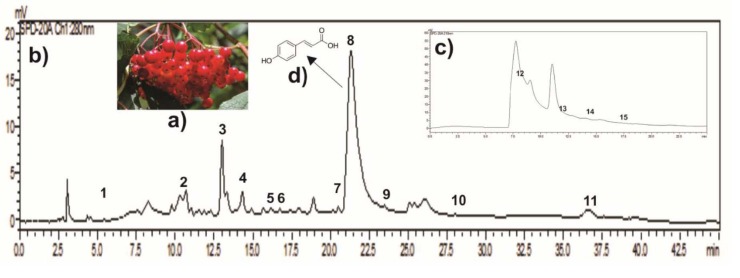
HPLC fingerprint profile of EVO. a) Images of *V. opulus* . b) Determination of phenolic compound content of EVO at 280 nm: 1. Gallic acid (6.33 min); 2. Protocatechuic acid (10.05 min); 3. Catechin hydrate (12.81 min); 4. 4-Hydroxybenzoic acid (14.09 min); 5. Caffeic acid (15.89 min); 6. Syringic acid (16.50 min); 7. Rutin trihydrate (20.24 min); 8. *p* -CA (21.19 min); 9. *Trans* -ferulic acid (23.20 min); 10. *Trans* -resveratrol (31.34 min); 11. Naringenin (36.41 min). c) Organic acid content of EVO at 210 nm: 12. Citric acid (9.02 min); 13. Maleic acid (12.5 min); 14. Formic acid (15.2 min); 15. Fumaric acid (18.6 min). d) Structure of *p* -CA.

*V.opulus* (Figure 1a) was subjected to methanol extraction, andthe phenolic content of EVO was determined by HPLC. Depending on the type of solvent, HPLC-UV chromatograms of EVO may show quite different profiles due to the structural dissimilarity of the solvents. When these solvents were compared, EVO displayed the highest amount of phenolic/flavonoid compounds as well as the highest antioxidant activity, while water and acetonitrile extracts of *V. opulus* possessed a low amount of phenolic compounds and lowantioxidant activity (Karacelik et al., 2015). The chromatogram of EVO after the derivatization is shown in Figures 1b and 1c. The peaks of phenolic compounds and organic acids were detected in the chromatogram, and the composition of EVOis listed in Table 2. As shown in Table 2, EVO contains a high amount of *p-* CA (280.5 ± 10.8 ppm) and catechin hydrate (235.9 ± 5.8 ppm), as well as citric acid (1198.1 ±16.9 ppm) and formic acid (349.6±5.1ppm). 

**Table 2:** Table 2. Phenolic and organic acid compositions of EVO identified by HPLC.

Peak number	RT (min)	Compound	The mean ± SD of concentration (ppm)
1	6.33	Gallic acid	0.8 ± 0.02
2	10.05	Protocatechuic acid	18.5 ± 1.90
3	12.81	Catechin hydrate	235.9 ± 5.80
4	14.09	4-Hydroxybenzoic acid	41.7 ± 3.40
5	15.89	Caffeic acid	5.2 ± 1.20
6	16.50	Syringic acid	3.4 ± 0.20
7	20.24	Rutin trihydrate	13.1 ± 0.20
8	21.19	Trans-p-coumaric acid	280.5 ± 10.80
9	23.20	Trans-ferulic acid	1.2 ± 0.05
10	31.34	Trans-resveratrol	0.4 ± 0.01
11	36.41	Naringenin	20.3 ± 1.20
12	9.02	Citric acid	1198.1 ± 16.90
13	12.5	Maleic acid	1.2 ± 0.20
14	15.2	Formic acid	349.6 ± 5.10
15	18.6	Fumaric acid	0.73 ± 0.10

SD: Standard deviation; RT: Retention time; ppm: Parts per million; min: Minutes.

Catechin hydrate significantly inhibited the proliferation of human cervical and breast cancer cell lines with IC_50_ values of 196.07 μg/mL and 127.62 μg/mL, respectively (Alshatwi, 2010; Al-Hazzani and Alshatwi, 2011).Citric acid,a well-known antioxidant compound, prevents the oxidation of macromolecules via scavenging reactive oxygen species (ROS). Citric acid treatment in the range of 400–1600 µg/mL was found to increase the cell apoptotic index (Chen et al., 2017). Karacelik et al. (2015) also showed that EVO possesses rutin, epicatechin, catechin, procyanidin B2, procyanidintrimer, and proanthocyanidin dimer monoglycoside. In addition, Velioglu et al. (2006) reported the concentrations of catechin and quercetin in EVO as 290.4 mg/kg and 52.1 mg/kg, respectively. *p-* CA possesses anticarcinogenic effects on human colon and cervical cancers and has been demonstrated to inhibit the proliferation of HT-29 and SW-480 cells (Sharma et al., 2018). Furthermore, EVO contains a limited amount of organic acids in which citric acid induces apoptosis via the mitochondrial pathway (Ying et al., 2013).

**Figure 2:**
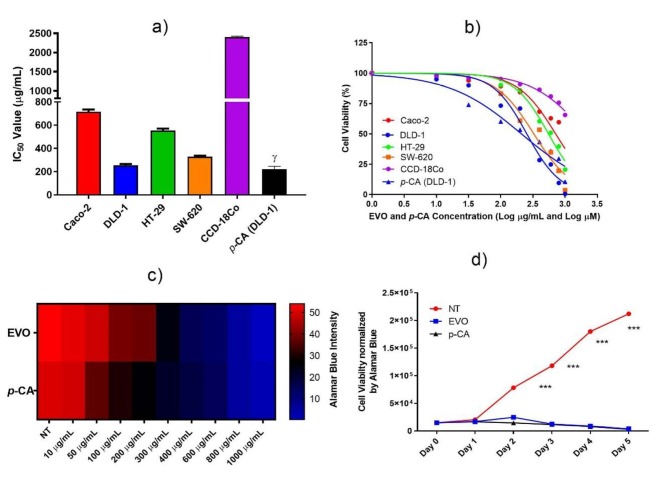
The effects of EVO and *p* -CA on the proliferation of human CRC cells; Caco-2, DLD-1, HT-29, SW-620 and healthy colon epithelium cell; CCD-18Co cells. a) Effects of EVO and *p* -CA on the proliferation of human CRC cells measured by Alamar Blue assay. b) IC_50_ values were determined sigmoidal plot of cell viability vs. log EVO (μg/mL) and log *p* -CA (μM) concentration. c) Heat map analyses of dose-dependent inhibition of EVO and p-CA against DLD-1 cells. Cell viability was decreased from red to blue color. d) Effects of EVO and *p* -CA on the growth of DLD-1 cells were monitored by automated cell counter. The results shown are representative of three independently performed experiments. NT: Nontreated; ***: P < 0.001; g: μM.

EVO was then evaluated for the antioxidant capacity using the DPPH and CUPRAC assays as shown in Table 3. The plant extract was found to have concentration-dependent inhibitory activity against DPPH radicals. The inhibition percentages of EVO on DPPH radical were 24.53 ± 0, 30.02 ± 0.34, 43.02 ± 1.74, 61.14 ± 1.98, and 89.99 ± 0.78 at 25, 50, 100, 200, and 400 µg/mL concentrations, respectively.The IC_50_ value for the DPPH radical scavenging capacity of EVO was 156.51 ± 1.12 µg/mL. Cupric ion reducing antioxidant capacity of the plant extract was determined as 44.06 ± 1.55 gallic acid equivalent (mg/g extract). Altun et al. (2008) investigated the antioxidant properties of the water extracts prepared from the different parts of *V. opulus* . Fruit extract showed DPPH scavenging activity with an IC_50_ value of 57 µg/mL. The total phenolic content was determined according to the equation obtained from the gallic acid standard curve(y=0.0074x+0.1064; R2=0.9957), whereas the total flavonoid content was calculated according to the equation obtained from the quercetin standard curve (mg/g extract) (y=0.0043x+0.0588; R2=0.9991). The total phenolic content of EVO was calculated as 310.07 ± 2.20 mg GAE/g extract, and the total flavonoid content was found to be 5.86± 0.40 mg QE/g extract. In a previous study, total phenolic contents of the fresh and pasteurized fruit juices of *V. opulus* were 351.26 ± 27.73 and 330.40 ± 29.46 mg GAE/100 mL, respectively (Cam and Hisil, 2007). Furthermore, Karacelik et al. (2015) investigated the antioxidant activity of the fruit juice and methanol, acetonitrile, and water extracts prepared from seeds and peels, separately. They found the highest activity in fruit juice (2.5-fold more than our result). In this study, we made methanol extract without separating the fruit parts; therefore, it is understandable that their results were higher than ours. After the determination of characteristic properties of EVO, we examined the antiproliferative activity of EVO and *p* -CA against human colorectal carcinoma cells (DLD-1, HT-29, SW-620, and Caco-2), and human colon epithelium cell (CCD-18Co). The Alamar Blue assay was performed, and the cells were treated with EVO and *p* -CA for 48 h. As shown in Figure 2a, EVO significantly and selectively inhibited proliferation of DLD-1, HT-29, SW-620, and Caco-2 cells, with IC_50_ values of 254.3 µg/mL, 553.3 µg/mL, 327.4 µg/mL, 714,6 µg/mL, respectively (P < 0.0001). EVO and *p* -CA inhibited the proliferation of DLD-1 and HT-29 cells in a dose-dependent manner. The genetic and molecular mechanisms in cancerous cells are different from each other. Therefore, these cells possess different behaviors against chemical agents. The cells of SW-620 are the strongest Wnt signal followers, while DLD-1 is the lowest. Maximum inhibitory action was found on DLD-1 cells. We investigated the antiproliferative effects of *p* -CA on this cell line, and the IC_50_ value of *p* -CA on DLD-1 cell was calculated as 223 µM (Figure 2b). It was also proven that *p* -CA inhibited HT-29 and Caco-2 cells with IC_50_ values of 1.5 mM and 1.6 mM, respectively (Jaganathan et al., 2013). On the other hand, the viability of healthy colon epithelium cells (CCD-18Co)were slightly inhibited after EVO and *p* -CA treatment in which IC_50_ was theoretically calculated (>2 mg/mL) from the sigmoidal plot as 2117 µg/mL and 1.6 mM, respectively (Figure 2b). Figures 2c and 2d show that EVO and *p* -CA have cytotoxic potential on the growth of DLD-1 cells and inhibited the proliferation of the cells in a dose-dependent manner. EVO contains a high amount of *p-* CA, which possesses carboxylic and phenolic OH groups, and catechin hydrate, which possesses phenolic OH groups. The carboxylic group is much more acidic than phenolic OH and may protonate the enzymes and DNA, whereas the phenolic OH makes a hydrogen bond with the enzymes and DNA. Bioflavonoids have broad anticancer characteristics based on their capability to promote differentiation and apoptosis, blockade of cellular carcinogen induction, cellular proliferation, tumor cell adhesion, and angiogenesis (De Kok et al., 2008). Some chemotherapeutic compounds have high cytotoxicity at low concentrations. However, these compounds have very limited utility because they can damage healthy cells as well as cancerous cells (Evdokiou et al., 2002; Beijers et al., 2012). Therefore, it is very important to determine the dose that affects cancerous cells but does not affect healthy cells in dose selection. The IC_50_ value of EVO was 254.3 µg/mL for human colorectal carcinoma cell line DLD-1, while it was more than 2 mg/mL for the healthy human colon epithelial cell line, CCD-18Co. Moderate cytotoxicity and safe concentration of EVO seem in balance for colorectal cancer treatment.

**Table 3:** Antioxidant capacity, total phenolic and flavonoid content of EVO.

Sample	DPPH radical scavenging capacity, the mean ± SD of the IC_50_ value (µg/mL)	CUPRAC value ± SD (mg GAE/g extract)	Total phenolic content ± SD (mg GAE/g extract)	Total flavonoid content ± SD(mg QE/g extract)
EVO	156.51 ± 1.12	44.06 ± 1.55	310.07 ± 2.20	5.86 ± 0.40
Quercetin	12.04 ± 0.87	-	-	-

SD: Standard deviation; GAE: Gallic acid equivalent; QE: Quercetin equivalent.

**Figure 3:**
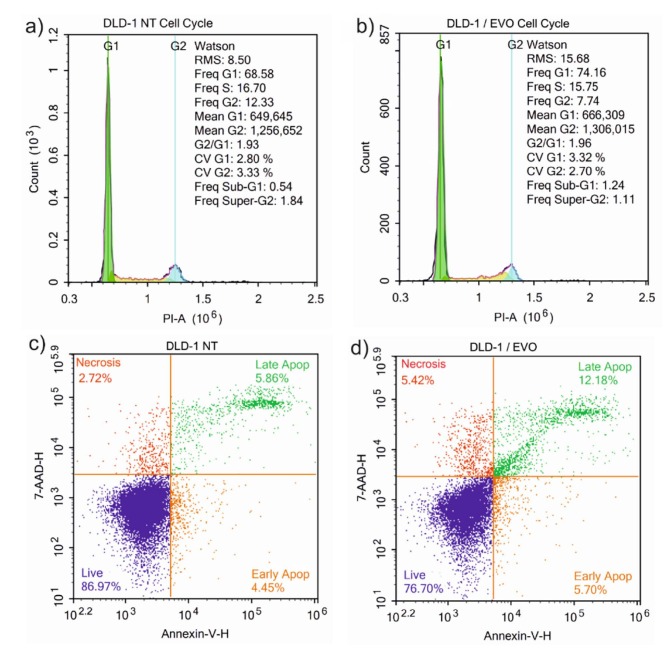
Representative cell cycle and apoptosis profile of DLD-1 cells determined by flow cytometer. a) and c) Cell cycle and apoptosis analyses of nontreated (NT) group, b) and d) EVO treated group (n = 3). DNA was stained with PI and 7-AAD for cell cycle and apoptosis analyses, respectively.

**Figure 4:**
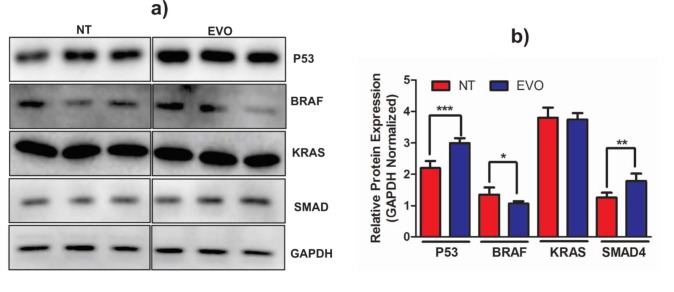
Effects of EVO on protein expression of key proteins in signaling pathways in colorectal carcinoma. a) Immunoblot bands of DLD-1 protein in nontreated (NT) and EVO groups. b) Quantitative band density analysis of the NT and EVO groups. Experiments were repeated at least 3 times (n = 6). (*: P = 0.0140), (**: P = 0.0011) and (***: P < 0.0001) signify a statistically significant difference.

To clarify the molecular mechanism of the inhibition effect of tumor cell (DLD-1) growth by EVO, the cell cycle and apoptosis were examined by ﬂow cytometry. Following treatment with 254 µg/mL of EVO for 48 h, the EVO groups exhibited statistically significant differences in cell cycle compared with the NT(nontreated) groups. A cell-cycle arrest at the G2/M checkpoint with an accumulation of cells in the G2 phase was observed. Following the treatment with EVO, the proportion of cells in the G2 phase and Freq Super-G2 decreased (1.7-fold) and the percentage of cells in Freq Sub-G1 increased significantly compared with the NT group (1.24% and 0.54%, respectively) (Figures 3a–3b). Increased Sub-G1 accumulation of DLD-1 cells confirmed the apoptosis. *p* -CA was also demonstrated to increase Sub-G1 and G2 phases in Caco-2 and HT-29 cells. Herein, we started to investigate the effects of EVO on apoptosis (Figures 3c–3d).The 48-h EVO treatment induced apoptosis 70% higher than in the control group. The apoptotic and necrotic rates in the NT group were as follows: necrosis 2.72%; late apoptosis 5.86%; early apoptosis 4.45%; live cells 86.97%. In the EVO-treated group, the rates were as follows: necrosis 5.42%; late apoptosis12.18%; early apoptosis 5.70%; live cells 76.70%. Thelate apoptosis of DLD-1 cells was significantly increased, which proved that the membrane integrity of the DLD-1 cell was disrupted and that the nucleus was released from the cell. It was also proven that *p* -CA treatment increased the apoptotic cell rate in DLD-1 cells. On the other hand, the apoptotic rates did not significantly change in HT-29 cells. To clarify the molecular mechanism of EVO on the proliferation of human colorectal carcinoma cells, we investigated the protein expression of key enzymes in signal transduction pathways including TGFβ, WNT, MAPK, PI3K-Akt, and p53 signaling pathways (Figure 4). Following the treatment of equivalent EVO concentration with IC_50_, protein expression of tumor suppressor p53and SMAD4 were significantly increased [1.43-fold (P < 0.0001) and 1.39-fold (P = 0.0011), respectively]. In CRC, the modulation of the p53 protein level is commonly seen; its landmark sign and relation with clinicopathological properties are still doubtful. Although some studies have reported no relationship between p53 protein levels and clinicopathological properties, some studies have indicated that p53 expression is related to the metastatic period (Aladhraei et al., 2019). Nevertheless, Cao et al. (2017) stated that the lack of p53 expression was reported in CRC tumors and related to a worse clinicopathological response ,including lymph node metastasis and progressive tumor development, because over 50% of human cancers present defects of function mutations in TP53gene. Hence, our results revealed that the protein expression of p53 in CRC cells was increased by the administration of EVO, which might provide a beneficial effect against CRC progression. The TGF-β1 induction promotes the activation of SMADs signalization. Previous studies have demonstrated that TGFβ signaling induced cancer cell proliferation, invasion, and metastasis, and blockade of TGF-β1 inhibits metastatic cancer development. Thus, the linkage of TGF-β1 inhibition that was used for the therapy of CRC has an immediate need to be clarified (Massague, 2008). According to our results, the protein expression of SMAD4 was found to be 1.39-fold higher than that of the control group. Jiang et al. (2018) reported that celastrol is an efficient drug which considerably prevented the mRNA and protein expression of SMAD4 in CRC cells. SMAD4, frequently deficient in CRC, has a role at the G1/S cell cycle in stopping cells waiting in the G1 cycle, hence resulting in cell cycle arrest (Zhao et al., 2018). Furthermore, the treatment of CRC cells with EVO resulted in higher mRNA and protein expression of SMAD4. This can be considered a protective effect of EVO. Thus, the elevation of SMAD4 induces CRC cells to stop the cell cycle on the G1 phase, which may lead to tumor suppression. BRAF has a pivotal function in cell trafficking as a positive modulator within the MAPK cascade. RAF is a member of the RAS––RAF–MEK–ERK cell-signaling cascade, with a central function in regulating cellular differentiation, proliferation, aging, and survival against extracellular indications (Caronia et al., 2011). BRAF expression was elevated in terminal stage CRC patients compared to early-stage CRC patients (P = 0.080) ( Kwon et al., 2018). It was found that pituitary adenomas possess the highest levels of BRAF mRNA and protein expressions (Ewing et al., 2007). Endometrial cells expressed 2.4-fold higher BRAF activity compared with the control cells and therefore caused abnormal activation of RAS/RAF/MAPK signaling (Yotova et al., 2011). The EVO treatment significantly decreased (21%; P = 0.014) the BRAF protein expression when compared with the control group. Thus, according to our findings, BRAF protein expression may be diminished in CRC cells via EVO. 

**Figure 5:**
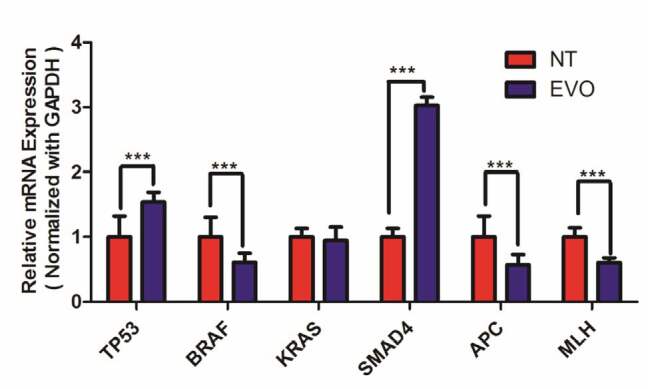
Effects of EVO on the mRNA expression of signal transduction key enzyme-coding genes; TP53, BRAF, KRAS, SMAD4, APC, and MLH of DLD-1 cells. Alterations in mRNA expressions were evaluated by qRT-PCR. Results were as relative mean ± SD (n = 4). As a housekeeping gene GAPDH were used. Fold change was calculated by 2^-ΔΔCt^. Groups were statistically analyzed with Student’s t-test. ***: P < 0.00.

To understand the origin of the alteration in the protein expressions, we investigated the mRNA expression of TP53, BRAF, KRAS, SMAD4, APC, and MLH genes (Figure 5). mRNA expression of tumor suppressor genes TP53 and SMAD4 were significantly (P < 0.0001) increased 1.54-fold and 3.03-fold, respectively. On the other hand, the mRNA expression of BRAF was significantly (P < 0.0001) decreased (41%) after the EVO treatment. Previous research has demonstrated that SMAD4 is profoundly related to the progression of colon metastases, and has a critical antimetastatic role in colon cancer metastasis (Yan et al., 2018). Sheng et al.(2019) reported that MiR-144 blocks the invasion of human colon cancer cells. The transfected SW620 colorectal carcinoma cells demonstrated lower SMAD4 expression. They also reported that SMAD4 is detected in 30%–40% of theCRC cases in the study, appearing in late stages in adenoma-to-carcinoma progression and resulting in liver metastases; these cases have a worse outcome with chemotherapy followed by a bad prognosis. In our study, SMAD4 was found to be elevated. The SMAD4 tumor suppressor gene product reduces TGF-β-mediated signaling and is defective in ~10% of CRC. Chang et al. (2019) concluded that with the help of targeted exome sequencing, higher deletion proportions of MSH3, MSH6, APC, and PIK3CA were found. They reported a significant role in CRC and found common deletions in RAF (9.38%), APC (59.38%), TP53 (50%), RAS (28.13%), and SMAD4 (9.38%).In addition to tumor suppressors and oncogenes, mRNA expression of DNA repair enzyme MLH1 and WNT signaling pathway regulator gene APC were also significantly inhibited (44% and 40%, respectively) with the EVO treatment. APC is commonly called a tumor suppressor gene, with a higher incidence of deletion in CRC. APC is disordered at both the germline and somatic steps (Zhang and Shay, 2017). The results of protein and mRNA expressions were evaluated together; the reason for the alteration of protein expressions seems to be the alteration in the mRNA expressions. 

## 4. Conclusion 

In this study, we clarify the molecular mechanism of the cytotoxic effects of medicinal herb *V. opulus* and its main active compound, *p* -CA, against human colorectal carcinoma. Both significantly inhibited the proliferation of human CRC line DLD-1, while no significant effects were observed against healthy colon cells. EVO and *p* -CA up-/downregulated CRC switch gene and protein expressions. Modulation of these genes affects 186 other genes which have crucial roles in TGF-β, MAPK, Wnt, and PI3K-Akt signaling pathways. Additionally, EVO and *p* -CA significantly increased apoptosis by altering Bax and Bcl-2. Therefore, EVO and its active compound *p* -CA may be a promising therapeutic agent against CRC.
